# Evaluation of the Impact of a Less-Invasive Trunk and Pelvic Trauma Protocol on Mortality in Patients with Severe Injury by Interrupted Time-Series Analysis

**DOI:** 10.3390/medicina60081338

**Published:** 2024-08-18

**Authors:** Tokiya Ishida, Yudai Iwasaki, Ryohei Yamamoto, Nozomi Tomita, Kazuaki Shinohara, Kaneyuki Kawamae, Masanori Yamauchi

**Affiliations:** 1 Department of Anesthesiology and Emergency Medicine, Ohta Nishinouchi Hospital, Fukushima 963-8558, Japan; 2 Department of Anesthesiology and Perioperative Medicine, Tohoku University Graduate School of Medicine, Sendai 980-8574, Japan; 3 Center for Innovative Research for Communities and Clinical Excellence (CIRC2LE), Fukushima Medical University, Fukushima 960-1295, Japan; 4 Department of Healthcare Epidemiology, School of Public Health, Graduate School of Medicine, Kyoto University, Kyoto 606-8501, Japan

**Keywords:** interrupted time-series analysis, minimally invasive trauma management, mortality, non-operative management, trauma

## Abstract

*Background and Objectives*: Minimally invasive trauma management, including interventional radiology and non-operative approaches, has proven effective. Consequently, our hospital established a trauma IVR protocol called “Ohta Nishinouchi Hospital trauma protocol (ONH trauma protocol) in 2013, mainly for trunk trauma. However, the efficacy of the ONH trauma protocol has remained unverified. We aimed to assess the protocol’s impact using interrupted time-series analysis (ITSA). *Materials and Methods*: This retrospective cohort study was conducted at Ohta Nishinouchi hospital, a tertiary emergency hospital, from January 2004 to December 2019. We included patients aged ≥ 18 years who presented to our institution due to severe trauma characterized by an Abbreviated Injury Scale of ≥3 in any region. The primary outcome was the incidence of in-hospital deaths per 100 transported patients with trauma. Multivariable logistic regression analysis was conducted with in-hospital mortality as the outcome, with no exposure before protocol implementation and with exposure after protocol implementation. *Results*: Overall, 4558 patients were included in the analysis. The ITSA showed no significant change in in-hospital deaths after protocol induction (level change −1.49, 95% confidence interval (CI) −4.82 to 1.84, *p* = 0.39; trend change −0.044, 95% CI −0.22 to 0.14, *p* = 0.63). However, the logistic regression analysis revealed a reduced mortality effect following protocol induction (odds ratio: 0.50, 95% CI: 0.37 to 0.66, *p* < 0.01, average marginal effects: −3.2%, 95% CI: −4.5 to −2.0, *p* < 0.01). *Conclusions*: The ITSA showed no association between the protocol and mortality. However, before-and-after testing revealed a positive impact on mortality. A comprehensive analysis, including ITSA, is recommended over before-and-after comparisons to assess the impact of the protocol.

## 1. Introduction

The current trend favors minimally invasive approaches in interventional radiology (IVR) and non-operative management (NOM) for severe trauma care [1,2]. In accordance with this change, several trauma training programs now highlight the importance of minimally invasive management [3]. It has been proved that IVR and NOM are not inferior in effectiveness to surgical treatment [4,5]. Thus, the potential circumvention of surgical intervention is a critical component in trauma management.

Therefore, in 2013, our institute initiated trauma treatment protocols named the “Ohta Nishinouchi Hospital trauma protocol (ONH trauma protocol)”, including less-invasive treatments such as IVR and NOM, as well as surgical management if needed, to standardize the management of patients with trauma and improve patient outcomes (Supplementary File S1). This trauma protocol was developed with a focus on trunk and pelvic trauma, where NOM may be effective, based on several scientific articles and publications [6,7,8,9,10,11,12,13].

Although implementing this protocol may improve the quality of multidisciplinary care and patient outcomes, a comprehensive evaluation of clinical outcomes has not been conducted since its implementation. Therefore, rigorous research is required to evaluate its efficacy in reducing mortality and increasing the number of IVR procedures performed.

Initially, these evaluations were conducted in a before-and-after study design [14], which has the advantage of a relatively simple design and requires little time and few resources. However, this design has poor internal validity, as it cannot exclude underlying trends as a cause for any change between two time points. For example, the quality of medicines and resuscitation before 2013 was estimated to be inferior to that from 2013 to 2019. Therefore, the effectiveness of protocol introduction should be evaluated while considering changing trends.

In contrast, interrupted time-series analysis (ITSA) uses multiple pre- and post-intervention observations to evaluate the effect of an intervention, and can account for underlying trends. ITSA has been widely used to evaluate healthcare interventions, including protocols for chronic disease management, behavioral interventions, and quality improvement initiatives [15].

This study aimed to assess the impact of our trauma management protocol using an ITSA, juxtaposing these findings with those obtained from a before-and-after comparative study design.

## 2. Materials and Methods

### 2.1. Study Setting and Protocol

This retrospective cohort study was conducted at the tertiary emergency center of Ohta Nishinouchi hospital (1086 beds, approximately 150 physicians) in Fukushima Prefecture, from January 2004 to December 2019. Koriyama is a region with a population of approximately 330,000 people, and the emergency department provides trauma care. Annually, our institution receives approximately 5000 patients through ambulance services, including 1100–1200 patients with trauma with varying degrees of severity.

The ONH trauma protocol was devised in 2012 and implemented in 2013. Focusing on non-operative management techniques such as NOM and IVR, this protocol ensures that trauma amenable to hemostasis through IVR receives such treatment. This has bolstered the quality of patient care for trauma cases presented to the emergency department, enabling optimal IVR practices. We formulated an educational and implementation guide emphasizing the importance of IVR for all emergency department physicians and medical personnel. The protocol was disseminated via lectures.

### 2.2. Study Population

This study included all patients aged ≥18 years who presented to our emergency department due to severe trauma characterized by an Abbreviated Injury Scale (AIS) score of ≥3 in any region [16]. The following categories were excluded: (1) patients transported more than 15 min after cardiac arrest, (2) patients suffering exclusively from head injuries, (3) patients presenting with burn or electrical shock injuries, and (4) patients with incomplete trauma-severity data.

### 2.3. Data Collection

Our hospital’s trauma database served as the primary data source. It provides an array of details, including age, sex, type of injury, AIS, Injury Severity Score (ISS) [17], Revised Trauma Score (RTS) [18], and Trauma and Injury Severity Score (TRISS) [19]. Additional factors encapsulated multiple traumas characterized by an AIS of ≥1 across two regions, the requirement for mechanical ventilation in the emergency department, utilization of medical response vehicles or air ambulances, and interventions administered upon admission. Intervention type was also recorded, including surgical procedures, abdominal surgery, thoracic surgery, and IVR. Lastly, survival status at the time of discharge was included.

### 2.4. Outcome Definition

Using our comprehensive trauma database, we identified cases of mortality in patients with trauma within our hospital from 1 January 2004 to 31 December 2019. We also determined preventable trauma deaths, characterized by in-hospital fatalities, despite a survival probability of >0.5 [20]. IVR was defined as procedures performed within the first 24 h after hospital arrival. The analysis employed data segmented into 3-month intervals as the time unit. The primary outcome was the incidence of in-hospital deaths per 100 transported patients with trauma, which effectively accounted for the patient volume ratio. As a secondary outcome, we evaluated the change in the number of IVR procedures per 100 patients with trauma per 3 months for patients with trauma before and after protocol implementation.

### 2.5. Statistical Analysis

We performed a descriptive analysis of the average age of patients with trauma, TRISS scores as measures of severity, in-hospital deaths, and IVR rates. For the ITSA analysis, the study period was divided into a pre-protocol implementation period (January 2004 to December 2011), a protocol development period (January 2012 to December 2012), and a post-protocol implementation period (January 2013 to December 2019). The protocol development period was considered the phase-in period. The Durbin–Watson test was used to evaluate autocorrelation. A segmental regression model without adjustment for autocorrelation was used to ensure the absence of apparent autocorrelation. The change in the number of in-hospital deaths per 100 patients with trauma every 3 months before and after the protocol implementation was evaluated. Trauma severity was included as a sensitivity analysis. As a secondary outcome, we assessed the change in the number of IVR procedures per 100 patients with trauma per 3 months for patients with trauma before and after protocol implementation.

Moreover, as a secondary analysis, individual data that did not consider changes over time were used. Multivariable logistic regression analysis was performed with in-hospital mortality or the proportion of preventable trauma death as the outcome, with no exposure before protocol implementation and with exposure after protocol implementation. As covariates, we adjusted for sex, age, type of injury, ISS, RTS, and mechanical ventilation in an emergency department. The average marginal effect (AME) of the protocol implementation was ascertained using the “margins” package in R, which calculates the change in the predicted probabilities for a one-unit change in the predictor variable, averaging over the distribution of the other covariates. Statistical analysis was performed using R 4.0.4 (The R Foundation for Statistical Computing Platform, Vienna, Austria). *p*-values < 0.05 were considered statistically significant.

## 3. Results

### 3.1. Patient Flow Chart and Descriptive Analysis

**Figure 1:**
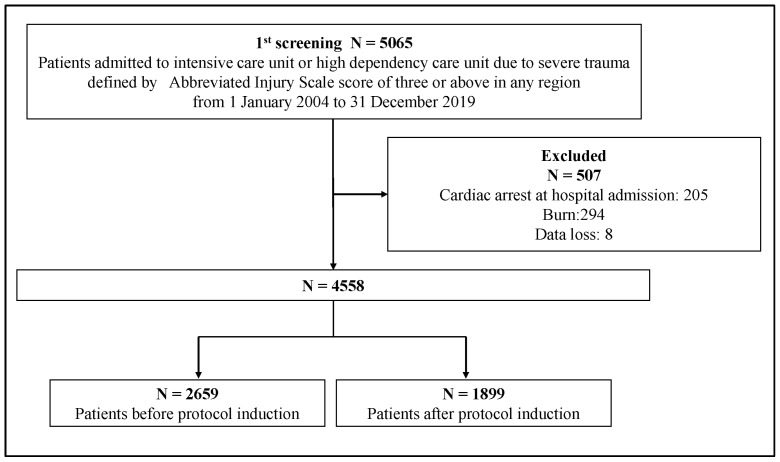
Flowchart.

**Figure 2:**
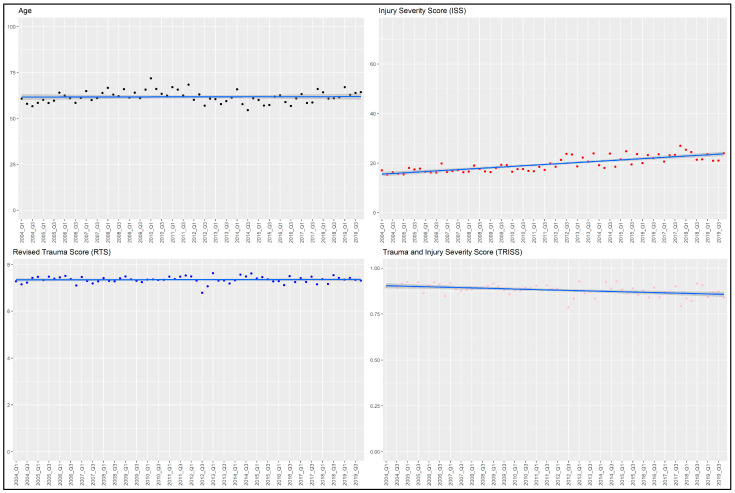
Baseline plot of mean age, mean ISS, mean RTS, and mean TRISS by calendar month. Q1: January to March, Q2: April to June, Q3: July to September, Q4: October to December. All data are represented as mean values. ISS: Injury Severity Score, RTS: Revised Trauma Score, TRISS: Trauma and Injury Severity Score.

In total, 5065 eligible patients were identified over the study period; 2659 patients were admitted before the trauma protocol induction, and 1899 patients were admitted after the trauma protocol induction. After the exclusion of 507 patients, 4558 patients were finally included in this study (Figure 1). Table 1 shows the baseline characteristics of patients in the before- and after-protocol groups. Compared with those in the before-protocol group, more patients in the after-protocol group had higher ISS and multiple traumas. Higher proportions of patients in the after-protocol group underwent mechanical ventilation in the emergency department and required medical response vehicles or air ambulances. Although TRISS was significantly higher in the after-protocol group (*p* < 0.001), it was not clinically different. Age, gender, and type of injury were also not clinically different between both groups. Figure 2 shows the baseline plot of mean age, mean ISS, mean RTS, and mean TRISS over 3 months. Mean ISS rose slightly every year, whereas TRISS decreased slightly with the changes in mean ISS.

### 3.2. Trends in the Study Period and ITSA Results

**Figure 3:**
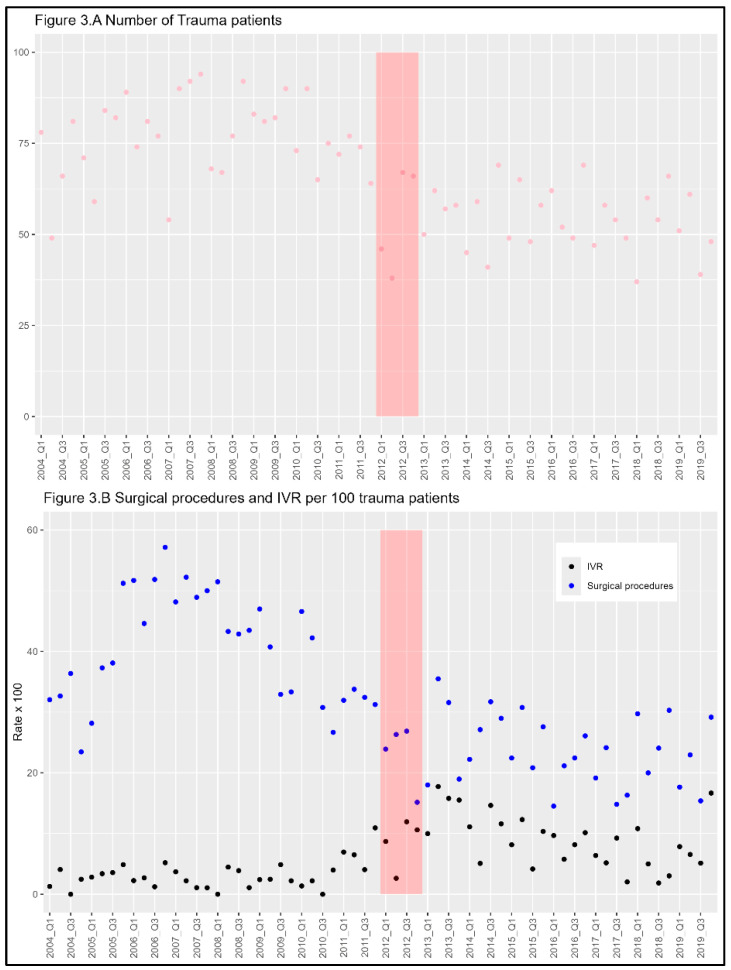
(**A**) Plot of the number of patients with trauma every month. (**B**) The number of IVRs per 100 patients with trauma every month. The pre-protocol implementation period was from 2004 Q1 to 2011 Q4; the protocol development period (phase-in period) was from 2012 Q1 to 2012 Q4, showing red shadow; the post-protocol implementation period was from 2013 Q1 to 2019 Q4. Q1: January to March, Q2: April to June, Q3: July to September, Q4: October to December; IVR: Interventional radiology.

**Figure 4:**
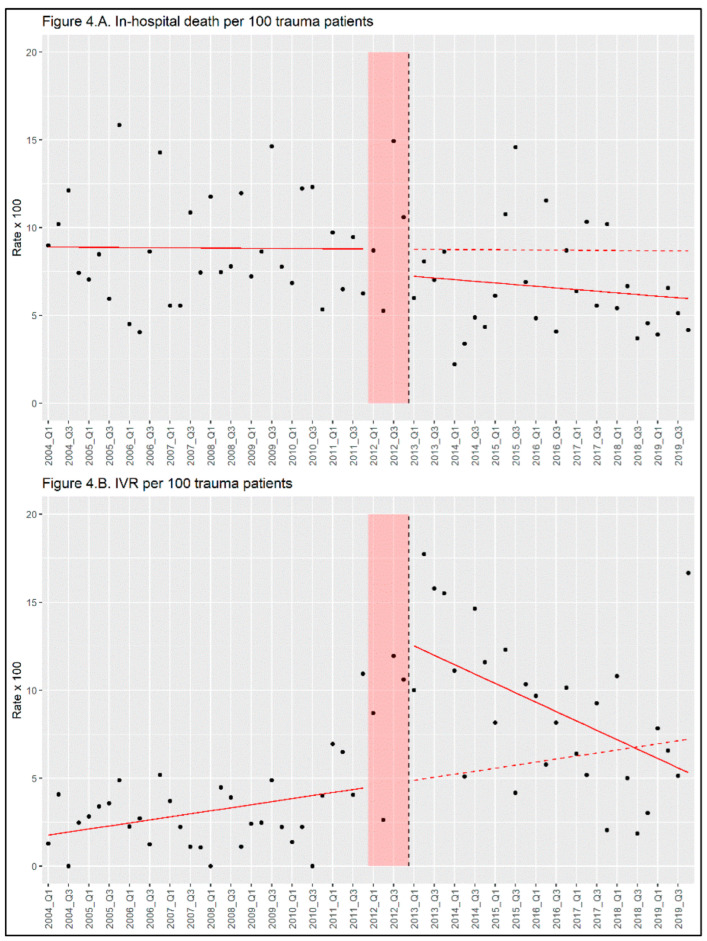
(**A**) The rate of in-hospital death per 100 trauma patients per 3 months. (**B**) The rate of IVR per 100 trauma patients per 3 months. The pre-protocol implementation period was from 2004 Q1 to 2011 Q4; the protocol development period (phase-in period) was from 2012 Q1 to 2012 Q4, showing red shadow; the post-protocol implementation period was from 2013 Q1 to 2019 Q4. The solid red line illustrates the trend for each observed period, whereas the dashed red line projects the continuation of the trend from the pre-implementation period. Q1: January to March, Q2: April to June, Q3: July to September, Q4: October to December. IVR: Interventional radiology.

Figure 3A shows the plot of the number of patients with trauma, which demonstrates the annual decrease in the number of injuries. Figure 3B shows the rates of IVR and surgical procedures per 100 patients with trauma. The number of surgical procedures gradually decreased during the study period. Figure 4A shows the mortality trends for patients with trauma over the study period, and Figure 4B depicts the number of IVR procedures per 100 trauma patients. As the slope shows, the mortality has declined in the after-protocol period compared with the before-protocol period. The number of IVR procedures increased drastically during the protocol development period and gradually decreased throughout the after-protocol period.

The Durbin–Watson test results showed no significant autocorrelation at any period in the study. Therefore, we proceeded with our ITSA without needing to correct for autocorrelation. Table 2 shows the outcomes of all the ITSA analyses. Before protocol induction, the death rate did not change over time (time change: −0.0036, 95% confidence interval [CI]: −0.12 to 0.11, *p*-value = 0.95). Immediate decrease (level change: −1.49, 95% CI: −4.82 to 1.84, *p*-value = 0.39) and decreasing trend (trend change: −0.044, 95% CI: −0.22 to 0.14, *p*-value = 0.63) did not show any significant decline. On sensitivity analysis, the protocol did not show a statistically superior impact. Figure 4B illustrates the trend in IVR per 100 patients over the study period. IVR per 100 patients increased initially but then decreased slowly (level change: 8.01, 95% CI: 4.48 to 11.53, *p*-value < 0.001, trend change: −0.35, 95% CI: −0.54 to −0.17, *p*-value < 0.001).

### 3.3. Secondary Analysis Using Individual Data: Before-and-After Comparative Study Design

Table 3 illustrates the comparison of clinical outcomes between the two groups. The proportion of operations or IVR increased in the after-protocol period. Mortality and the proportion of preventable trauma deaths were lower in the after-protocol group than in the before-protocol group.

Logistic regression analysis showed that the induction of the protocol was associated with a reduction in both mortality (odds ratio: 0.50, 95% CI: 0.37 to 0.66, *p*-value < 0.01, AME: −3.2%, 95% CI: −4.5 to −2.0, *p*-value < 0.01) and the proportion of preventable trauma deaths (odds ratio: 0.67, 95% CI: 0.36 to 0.69, *p*-value = 0.02, AME: −1.4%, 95% CI −2.5 to −0.3, *p*-value = 0.01).

## 4. Discussion

In this study, ITSA did not demonstrate an association between the trauma protocol and in-hospital death. We observed a younger age demographic, increased proportion of male patients, and greater trauma severity in the post-protocol group compared to the pre-protocol group. These results may be associated with differences in trauma severity between sexes [21] and changes in emergency medicine systems within this study region. During the post-protocol study period, the centralization of severely injured patients at our hospital led to an increase in trauma severity, which in turn influenced the observed sex differences. Autocorrelation was not detected in our analysis, and the mortality trends before and after protocol induction did not differ. Sensitivity analysis, which adjusted for trauma severity as the most important covariate, revealed a similar result to the original analysis. Although the age differences were not statistically significant, the proportion of male patients and trauma severity were significantly different between the groups. According to a secondary analysis of the before-and-after comparison via multivariable logistic regression analysis, which includes these covariates, the use of ONH trauma protocol was associated with in-hospital mortality and the proportion of preventable trauma death.

This indicates that IVR-driven procedures in severe trauma management do not cause significant harm. Our 95% CI for the effect of protocol implementation was −4.82 to 1.84, indicating a small effect or small possible harm. In our results, surgical procedures decreased in the after-protocol period compared to the before-protocol period, and protocol introduction was associated with an increase in IVR, which was consistent with the results of a previous study [22]. However, a drastic increase in the number of surgical procedures was observed during the protocol development period, while a decline in the number of IVR procedures was observed during the after-protocol period. This phenomenon might reflect an initial overreaction [23], after which appropriate procedures were selected when managing trauma cases. The introduction of specific protocols may facilitate safe hemorrhage control, potentially obviating the need for surgical interventions. Despite several differences in factors between the two groups, the sophisticated statistical design of ITSA and the sensitivity analysis adjusted for trauma severity consistently indicated similar outcomes. According to the results of these analyses, the ONH trauma protocol was implemented without incurring any significant harm. Conversely, the before-and-after comparison test revealed an association between the implementation of the trauma protocol and in-hospital mortality. This discrepancy warrants careful consideration and highlights the importance of selecting an appropriate analytical approach.

Some studies have verified the effects of trauma protocol implementation by using before-and-after study comparisons [24,25], and these analyses indicated preferable results for each protocol. The before-and-after comparison test is a straightforward method of determining the effect of an intervention. However, its result might be affected by regression to the mean [26], and this study design also cannot adequately control for possible confounding factors that may contribute to changes in outcomes [27,28].

Compared with the before-and-after comparison, ITSA is a robust method that accounts for underlying trends, seasonal factors, and autocorrelation [15,29]. This statistical method is commonly used in health services research, especially when randomization of intervention is not applicable [15]. It considers the temporal structure of the data and adjusts for the possibility of a trend that may exist, even in the absence of the intervention. Hence, ITSA could provide a more realistic picture of the effectiveness of an intervention implemented over time, as it considers the pre-existing trend and the time required for the intervention to achieve its full effect. The time trend for mortality may differ because trauma management is improving year after year in Japan [30]. Moreover, the randomization of intervention was not applicable in this study. In this setting, a before-and-after study comparison may distort the results.

The difference in the results between the ITSA and the before-and-after comparison may be indicative of the different statistical characteristics of these two methods. In addition, changes in trends may have influenced the analysis. In this cohort, the annual number of injuries decreased every year, which is consistent with a decline in traffic accidents and a significant decrease in fatal accident rates in Japan that was reported previously [31]. The 95% CI for the effect on in-hospital deaths ranged from −4.90 to 1.92 per 100 patients with trauma; therefore, a small effect could not be ruled out. Although before-and-after comparisons provide some insights into the impact of an intervention, they have significant limitations, which may yield results that are inconsistent with those of ITSA. Given its capability to account for temporal trends and external influences more accurately, ITSA offers a more robust method for evaluating the true impact of protocol implementation. Therefore, rather than relying solely on before-and-after comparisons, it is advisable to consider the more precise ITSA during the assessment of intervention outcomes.

### Limitations

This study has a few limitations. First, the difference in patients’ characteristics in each period may affect the ITSA results, although we conducted a sensitivity analysis that adjusted for trauma severity. Second, we could not collect data on the time until the computed tomography scan and the time until IVR or the operation, which might have been affected by the protocol induction, and should be evaluated. Our trauma database did not include such data, and we could not verify the effects of the protocol in terms of the quality of trauma care. Although our trauma IVR protocol did not have a positive effect on patient mortality, it may have improved trauma management. Moreover, this study highlights the importance of selecting appropriate statistical analyses.

## 5. Conclusions

The ITSA did not illustrate the trauma protocol’s effectiveness, although before-and-after comparison tests showed a statistically significant reduction in mortality. The difference in analytical characteristics may cause these discrepancies, and our findings highlight the importance of using appropriate analytical methods while evaluating the effectiveness of interventions over time.

## Figures and Tables

**Table 1:** Comparison of the baseline characteristics of patients between the two groups.

	All Patients	Before Protocol	After Protocol	
Patients’ Characteristics	n = 4558	n = 2659	n = 1899	*p*-Value
Age, years, median (IQR)	66 (48–78)	67 (49–79)	64 (48–77)	0.008
Male, n (%)	2942 (64.5)	1614 (60.7)	1328 (69.9)	<0.001
Type of injury (%)				
Crash	638 (14.0)	331 (12.4)	307 (16.2)	NA
Fall	204 (4.5)	92 (3.5)	112 (5.9)	
Traffic accident	2062 (45.2)	1100 (41.4)	962 (50.7)	
Other trauma	1654 (36.3)	1136 (42.7)	518 (27.3)	
ISS, median (IQR)	16 (9–25)	13 (9–24)	19 (13–29)	<0.001
RTS, median (IQR)	7.84 (7.55–7.84)	7.84 (7.55–7.84)	7.84 (7.55–7.84)	0.014
TRISS, median (IQR)	0.97 (0.91–0.97)	0.97 (0.93–0.97)	0.96 (0.89–0.97)	<0.001
Abdomen AIS, median (IQR)	0 (0–1)	0 (0–0)	0 (0–2)	<0.001
Pelvic AIS, median (IQR)	2 (1–3)	2 (1–3)	1 (1–3)	<0.001
Thoracic AIS, median (IQR)	0 (0–3)	0 (0–3)	3 (0–4)	<0.001
Multiple trauma, n (%)	1170 (25.7)	548 (20.6)	622 (32.8)	<0.001
Mechanical ventilation at the emergency department, n (%)	681 (14.9)	353 (13.3)	328 (17.3)	<0.001
Use of medical response vehicle or air ambulance, n (%)	794 (17.4)	378 (14.2)	416 (21.9)	<0.001

IQR: interquartile range, ISS: injury severity score, RTS: revised trauma score, TRISS: trauma and injury-severity score, AIS: abbreviated injury scale.

**Table 2:** Outcomes of ITSA analyses.

	Coefficient	95% CI	*p*-Value
Impact of the protocol on in-hospital deaths (primary outcome)			
Time change	−0.0036	−0.12 to 0.11	0.95
Level change	−1.49	−4.82 to 1.84	0.39
Trend change	−0.044	−0.22 to 0.14	0.63
Impact of the protocol on in-hospital deaths (according to sensitivity analysis of the primary outcome)			
Time change	−0.035	−0.14 to 0.067	0.50
Level change	−1.18	−4.15 to 1.79	0.44
Trend change	−0.08	−0.239 to 0.079	0.33
TRISS	−54.1	−81.0 to −27.2	<0.001
Impact of the protocol on the number of IVR procedures (secondary outcome)			
Time change	0.09	−0.03 to 0.21	0.151
Level change	8.01	4.48 to 11.53	<0.001
Trend change	−0.35	−0.54 to −0.17	<0.001

ITSA: interrupted time-series analysis, CI: confidence interval, TRISS: trauma and injury severity score.

**Table 3:** Comparison of clinical outcomes between the two groups. IVR: interventional radiology.

	All Patients	Before Protocol	After Protocol	
Clinical Outcomes	n = 4558	n = 2659	n = 1899	*p*-Value
Death, n (%)	361 (7.9)	240 (9.0)	121 (6.4)	0.001
Emergent IVR, n (%)	237 (5.2)	70 (2.6)	167 (8.8)	<0.001
Operation or IVR on the day of admission (%)	1703 (37.4)	1170 (44.0)	533 (28.1)	<0.001
Preventable trauma deaths, n (%)	179 (3.9)	113 (4.2)	66 (3.5)	0.001

## Data Availability

The data that support the findings of this study are available from the corresponding author upon reasonable request.

## Supplementary Materials

The following supporting information can be downloaded at: https://www.mdpi.com/article/10.3390/medicina60081338/s1, Supplemental File S1: Summary of Ohta Nishinouchi Hospital trauma protocol.
