# Supplementary material for: Evaluation of the Impact of a Less-Invasive Trunk and Pelvic Trauma Protocol on Mortality in Patients with Severe Injury by Interrupted Time-Series Analysis
Source: Medicina (Kaunas). 2024 Aug 18;60(8):1338. doi: 10.3390/medicina60081338 (PMC11356191; doi:10.3390/medicina60081338)

Supplemental File S1
Summary of trauma protocol at Ohta Nishinouchi hospital

<u>Abbreviations</u>
AAST-OIS: Association for the Surgery of Trauma-Organ Injury Scale
CT: Computed tomography
DLT： Double lumen tube
DP: Distal pancreatectomy
ER: Emergency room
ERP: Endoscopic retrograde pancreatography
EV: Extravasation
IABO: Intra-aortic balloon occlusion
IVR: Interventional Radiology
JAST: Japanese Association for the Surgery of Trauma
OR: Operating room
PD: Pancreaticoduodenectomy
PRD: Perirenal hematoma rim distance
SLT： Single lumen tube

[Goal and Principle]
CT scan: within 30 minutes after hospital arrival
IVR: within 45–60 minutes after hospital arrival
IABO application: hemorrhagic shock, defined as a mean blood pressure of less than 60 mmHg, is prolonged despite the appropriate therapy.

[Thoracoabdominal trauma]
Adaptation of emergent laparotomy
1. Free air detected by CT scan
2. Mesenteric injury
3. Non-responder despite adequate fluid therapy (including trauma cases with pelvic fracture）
4. Penetrating trauma (consider checking diaphragm injury)

1. Protocol for liver injury

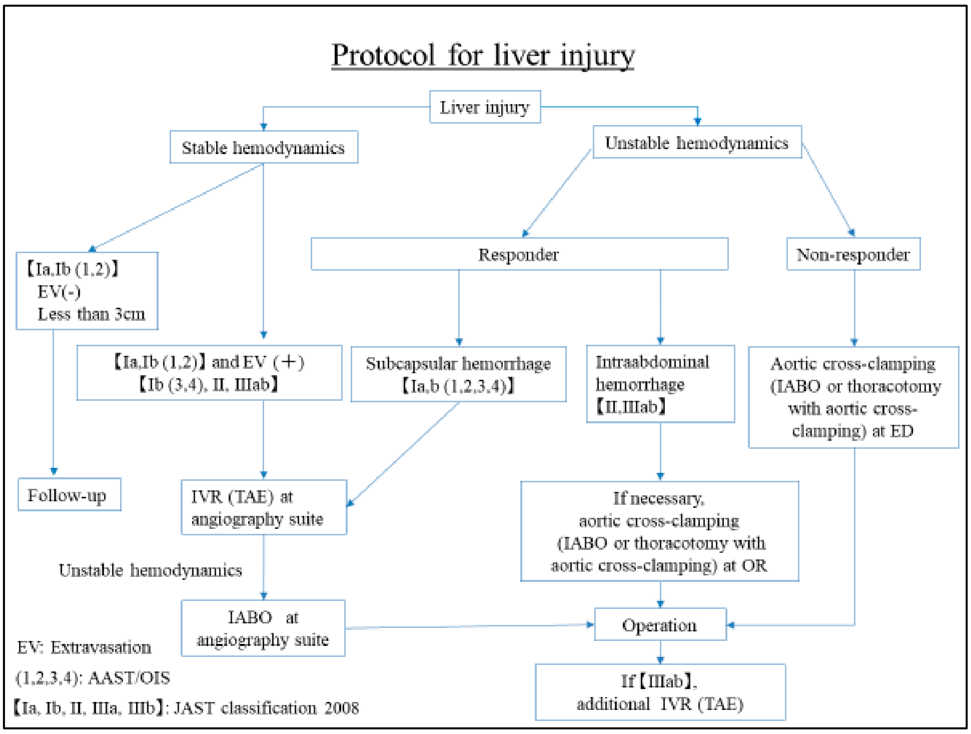

2. Protocol for spleen injury

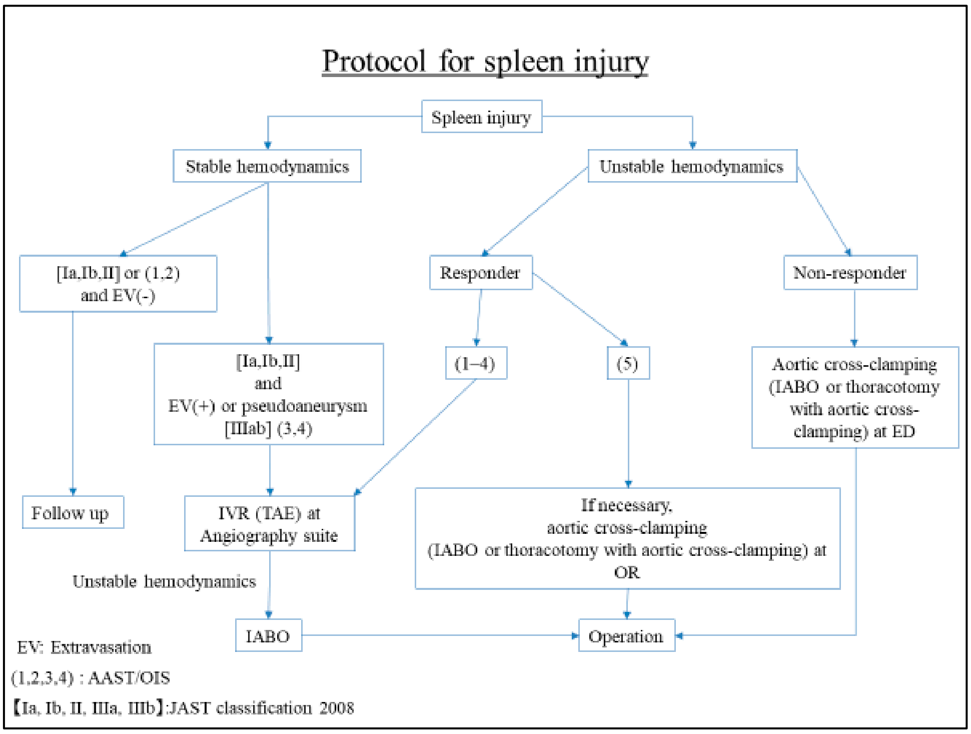

3. Protocol for kidney injury

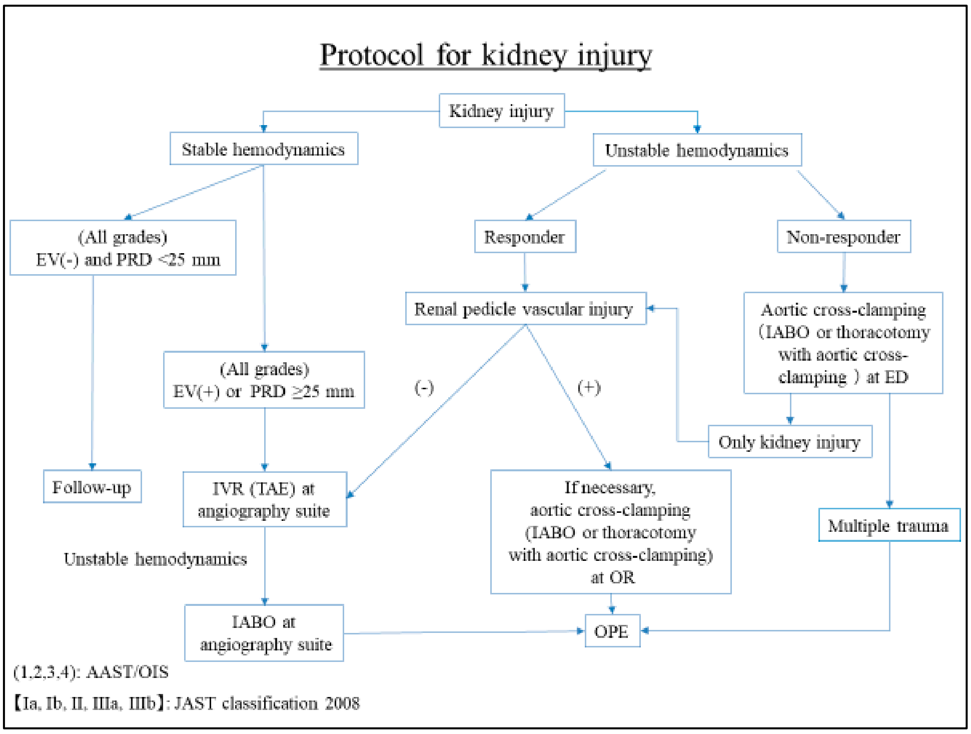

4. Protocol for pancreas injury

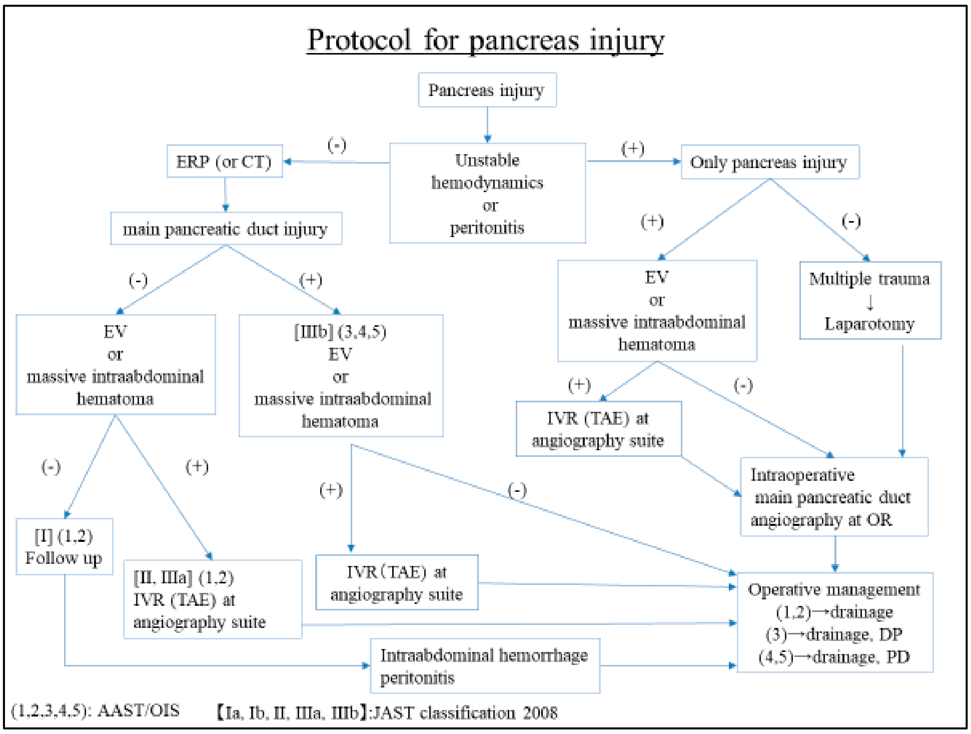

5. Protocol for blunt lung injury and rib fractures

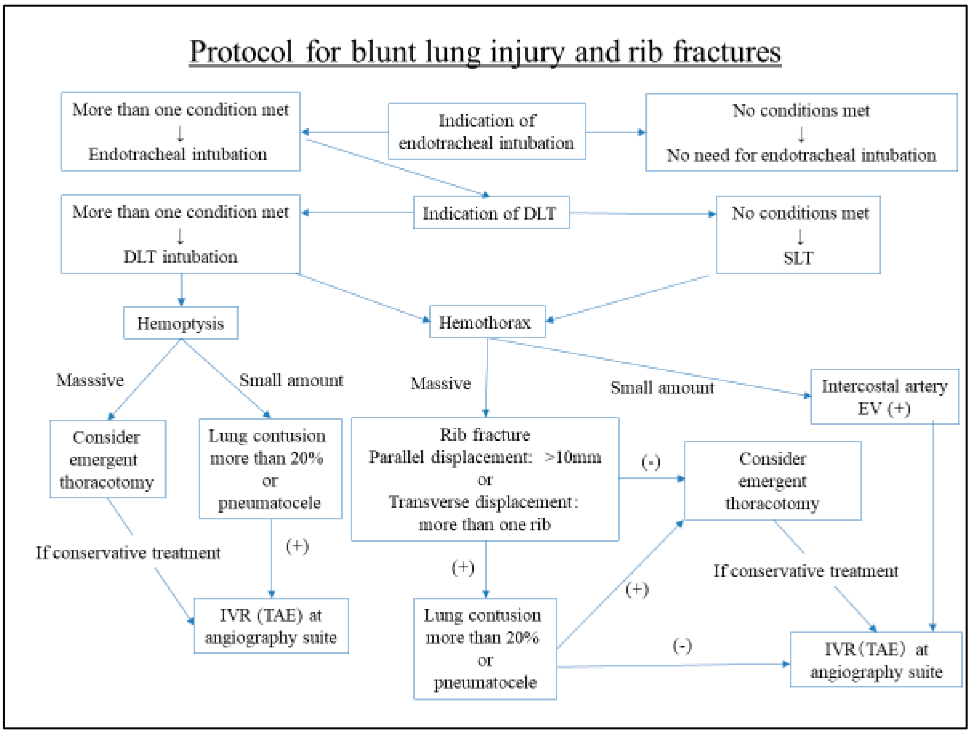

*1: Indications for Endotracheal Intubation (any one of No.1–4 applies)

No.1: Hypoxemia requiring mechanical ventilation, hypercapnia
(Consider Non-Invasive Positive Pressure Ventilation (NPPV) if only No.1 applies)

No.2: Abnormalities in the airway (such as accompanying facial injuries)

No.3: When hemoptysis is suspected

No.4: When the following conditions are met:
-Glasgow Coma Scale (GCS) <14
-Pulmonary contusion covering more than approximately 30% of the total lungs
-Rib fractures: 5 or more
All 3 conditions met → endotracheal intubation is necessary
1–2 conditions met → consider endotracheal intubation
No conditions met → no need for endotracheal intubation

6. Protocol for diaphragm injury

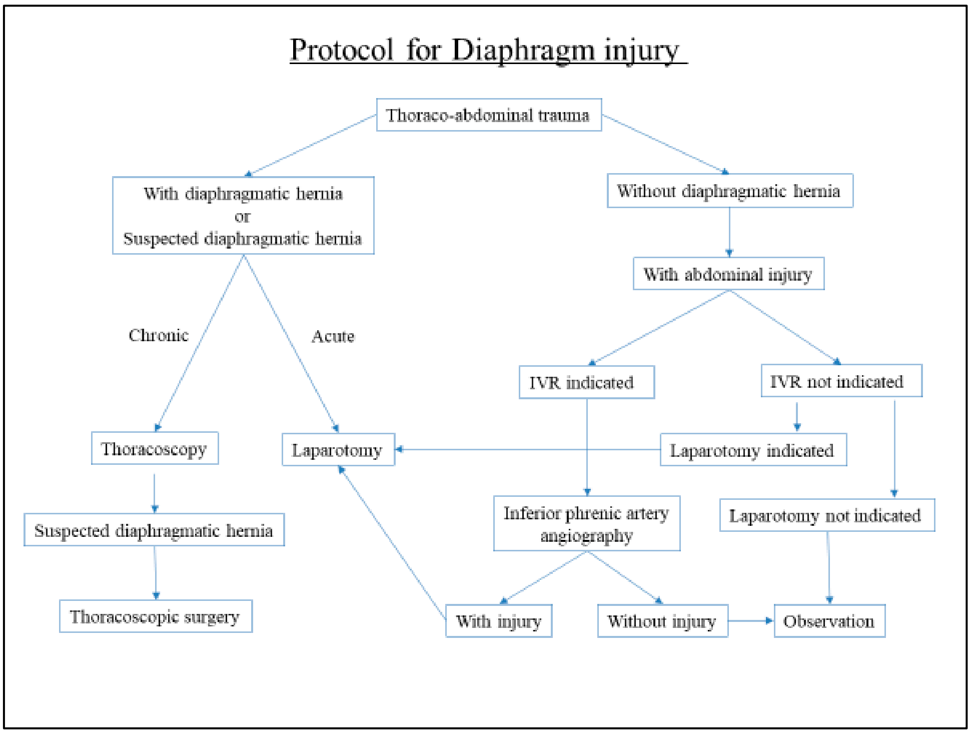

7. Protocol for pelvic fracture

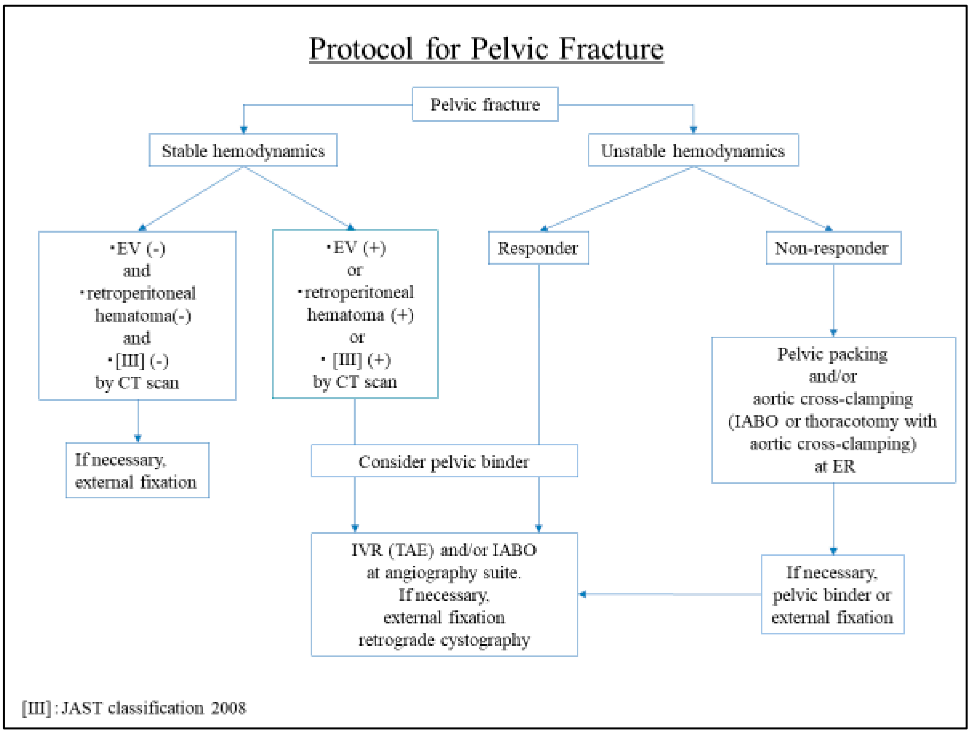

Supplement: Supplementary file 1 [file medicina-60-01338-s001.zip › medicina-3152212-supplementary.pdf]
